# Establishing a critical care network in Asia to improve care for critically ill patients in low- and middle-income countries

**DOI:** 10.1186/s13054-020-03321-7

**Published:** 2020-10-15

**Authors:** CRIT CARE ASIA, Abi Beane, Arjen M. Dondorp, Arshad Taqi, A. S. M. Areef Ahsan, Bharath Kumar Tirupakuzhi Vijayaraghavan, Chairrat Permpikul, Christopher Pell, David Gandy, Dilanthi Priyadarshani, Diptesh Aryal, Dong Phu Khiem, Duong Bich Thuy, Guy Thwaites, Gyan Kayastha, Ishara Udayanga, Jorge Salluh, Khamsay Detleuxay, Lakshmi Ranganathan, Lam Minh Yen, Lim Chew Har, Louise Thwaites, Madiha Hashmi, Marcus J. Schultz, Mavuto Mukaka, Meghan Leaver, Mohd Basri Mat Nor, Muhammad Hayat, Nick Day, Ramani Moonesinghe, Rashan Haniffa, Ratapum Champunot, Rebecca Inglis, Rozina Sultana, Sophie Yacoub, Steve Harris, Subhash Prasad Acharya, Swagata Tripathy, Syed Muneeb Ali, Tamilarasu Kadhiravan, Yoel Lubell

**Affiliations:** Collaboration for Research, Implementation and Training in intensive CARE in ASIA, Mahidol Oxford Tropical Research Unit, 3/F, 60th Anniversary Chalermprakiat Building, 420/6 Rajvithi Road, Bangkok, Thailand

**Keywords:** Critical care, Quality improvement, Registry, Low- and middle-income countries

## Introduction

When undertaking quality improvement (QI) initiatives, one of the greatest burdens is repeated data collection. Intensive care registries, such as those commonly used in high-income countries (HICs), have enabled systematic capture of routine information needed to measure intensive care unit (ICU) performance [1]. Once considered unfeasible in resource-limited settings, newer cloud-based platforms are gaining increasing traction. Collaborative surveillance platforms, such as NICS-MORU and PRICE, which have mobile and desktop applications, have established methods for daily capture of individual patient-level information and have shown that—even in resource-limited settings—the systematic evaluation of patient care throughout the hospital journey is feasible at scale using coalesced minimal data sets [2, 3].

## Quality of care

Poor quality of care has resulted in an estimated additional five million deaths, and six trillion US dollars in economic losses worldwide [4, 5]. In South and South-East Asia, a region which accounts for over 25% of the world’s population, poor quality health care is one of the biggest drivers of excess morbidity and mortality [4]. Recent recommendations from the Lancet Global Health Commission have called for greater investment in systems that strengthen evaluation and improvement, and a focus on healthcare that is reflective of and sensitive to the diverse needs of communities [5].

Critical care is expensive and complex. Many barriers impede the optimal care of critically ill patients, especially in resource-restricted settings [6]. Basic equipment for monitoring, treatment and diagnosis is often unavailable and maintenance is suboptimal [7]. Supplies of laboratory consumables and essential medications can be unpredictable and the provision of basic commodities, such as oxygen, electricity and running water, unreliable. Despite these challenges, demand for these services continues and with it the need to establish systems by which quality of care can be continually improved.

## Barriers to quality improvement in LMICs

### Lack of information for quality evaluation

The ability to continually evaluate care and empower stakeholders to identify priorities for improvement is a crucial but missing component of QI [4, 8]. In low- and middle-income countries (LMICs), the lack of reliable facility-level and national information has hampered the implementation of QI initiatives and prevented clinicians from identifying local research priorities [2].

### Limited success of quality improvement

Many of the basic principles of ‘good quality’ critical care that have proved successful in HICs may be directly applicable to resource-limited settings. Often, however, practices are poorly implemented. To date, in LMICs, QI initiatives have had limited success in achieving sustained change or have proven difficult to scale [5]. Quality improvement methods are generally neither an established part of medical education nor are they a priority investment for healthcare institutions in resource-limited settings [5, 9].

## A pathway for improving quality of care

**Fig. 1:**
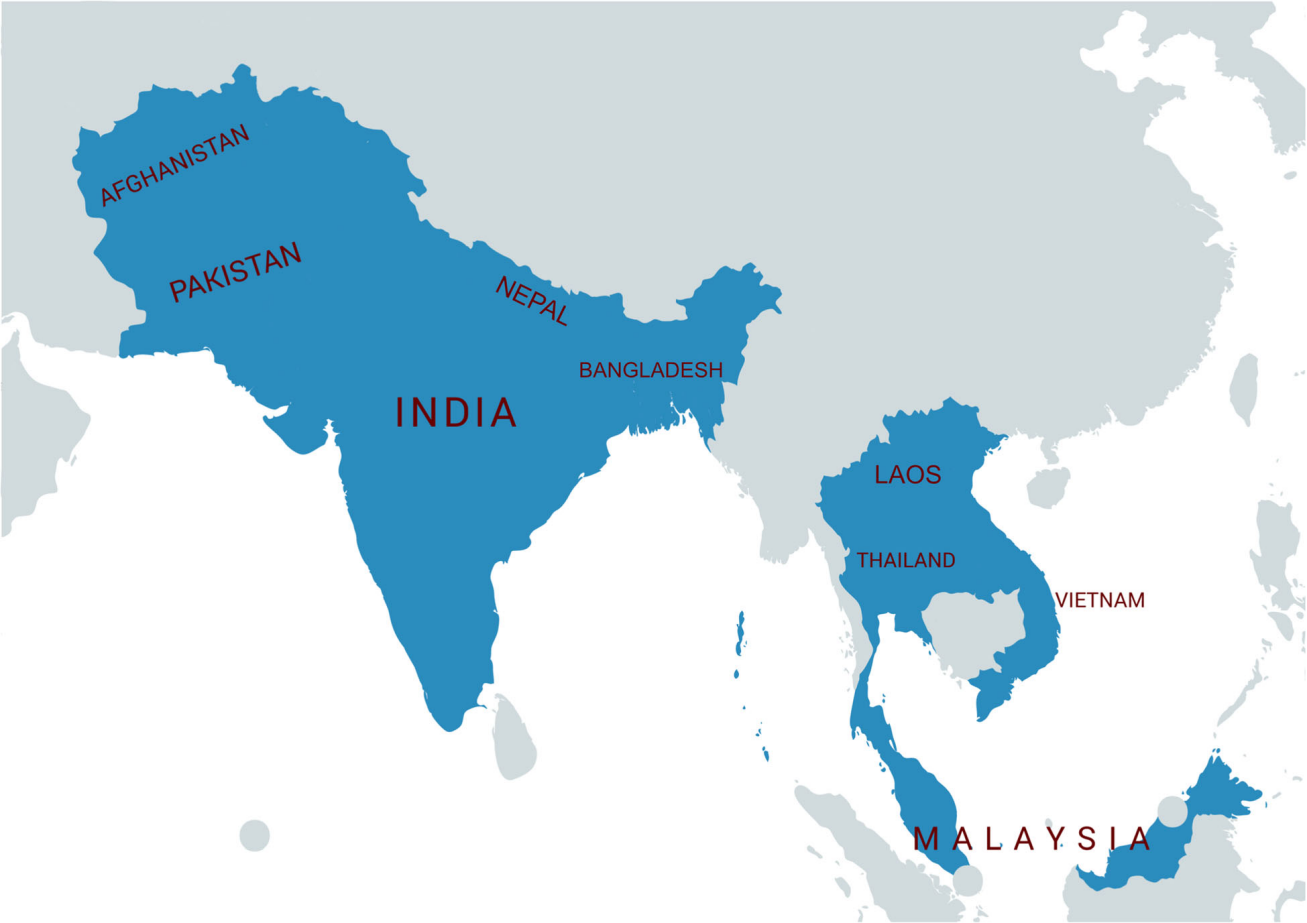
Countries participating in the CRIT CARE ASIA network: Afghanistan, Bangladesh, India, Laos, Malaysia, Nepal, Pakistan, Thailand and Vietnam. Figure created using MapChart under the CC BY-SA 4.0 license [10].

Supported by a Wellcome Innovations Flagship Programme grant, our group of multidisciplinary healthcare professionals predominantly based in Asia, is establishing a locally led collaborative network: Collaboration for Research, Implementation and Training in intensive CARE in ASIA (CRIT CARE ASIA). The collaboration will improve patient outcomes using near real-time high-quality data to drive improvement and strengthen the health system through a system of audit and feedback. Delivered over 3 years and extendable beyond this period, the programme will establish an Asian ICU network across 42 units in nine countries and implement a setting-adapted electronic cloud-based registry co-designed and developed by clinicians in the region (Fig. 1). Using the registry, plus qualitative and quantitative research methods, CRIT CARE ASIA will evaluate the quality of critical care, which will then lead into locally led QI interventions to improve ICU performance and patient outcomes driven by the priorities of stakeholders.

### Establishing a collaborative network

Initiating, maintaining or advancing a local improvement programme consumes large amounts of time and energy [11]. Establishing or joining a collaboration for shared improvement, such as CRIT CARE ASIA, may help. Collaborative improvement networks engage clinicians, researchers, patients and policy-makers to test approaches to improve care, translate research and prioritise service delivery [2, 3]. CRIT CARE ASIA will connect ICUs and institutions to provide diverse high-quality data using an agreed core dataset to generate evidence and inform clinical decision-making. The network uses collaboration, data science, clinical training and implementation science methods pragmatically adapted to resource-limited settings. These combined methods create a feedback loop within the ICU and allow operational problems to be quickly corrected and potential pitfalls of implementation of the QI intervention to be avoided.

### Utilise a problem-solving approach to quality improvement

To improve quality of care, it is necessary to locate the gap in care and to identify and understand its underlying determinants. Utilising mixed-methods, the network will encourage a *learning by doing model*, whereby members of the network will be supported to interact with data generated through the registry and identify problems and possible solutions. Qualitative approaches, including ‘real-time evaluations’, from disciplines including anthropology and business, which lend themselves to rapid evaluation of complex health systems, will be utilised [12]. Findings from interviews, observations and focus group discussions will inform the design and implementation of targeted QI projects.

### Challenges

The greatest challenge lies with information governance aspects of data curation and sharing. The diverse project team will use their extensive LMIC experience to overcome barriers to data sharing. International research partnerships can disproportionately advantage high-income countries and institutions [13]. With this in mind, we have proactively incorporated measures including inclusive priority-setting and locally led improvement into the design of the network activities.

## Future

The network will enable the implementation and evaluation of innovative LMIC technologies and provide opportunity for data linkage with the registry [14]. The ICU network, supported by the electronic registry, will facilitate epidemiological and clinical research. CRIT CARE ASIA uses a Common Data Model and standard nomenclature and coding, which will facilitate comparison of data with globally relevant health data networks such as ISARIC and LOGIC. Many critically unwell patients are cared for outside of the ICU [6]. Many of the strategies to improve the quality of processes of care will therefore need to extend beyond the ICU to encompass surgical, emergency medicine and community-based services as already demonstrated in Sri Lanka [15].

## Data Availability

Data sharing is not applicable to this article as no datasets were generated or analysed during the current study.

## Abbreviations

CRIT CARE ASIA: Collaboration for Research, Improvement and Training in Critical CARE in ASIA
HIC: High-income country
ICU: Intensive care unit
LMIC: Low- and middle-income country
QI: Quality improvement
